# Geometric and network organization of visceral organ epithelium

**DOI:** 10.3389/fnetp.2023.1144186

**Published:** 2023-05-10

**Authors:** Betty S. Liu, Joseph Sutlive, Willi L. Wagner, Hassan A. Khalil, Zi Chen, Maximilian Ackermann, Steven J. Mentzer

**Affiliations:** ^1^ Laboratory of Adaptive and Regenerative Biology, Brigham and Women’s Hospital, Harvard Medical School, Boston, MA, United States; ^2^ Translational Lung Research Center, Department of Diagnostic and Interventional Radiology, University of Heidelberg, Heidelberg, Germany; ^3^ Institute of Functional and Clinical Anatomy, University Medical Center of the Johannes Gutenberg-University, Mainz, Germany

**Keywords:** pleura, epithelial cells, connectivity (graph theory), cell size, visceral organ

## Abstract

Mammalian epithelia form a continuous sheet of cells that line the surface of visceral organs. To analyze the epithelial organization of the heart, lung, liver and bowel, epithelial cells were labeled *in situ*, isolated as a single layer and imaged as large epithelial digitally combine montages. The stitched epithelial images were analyzed for geometric and network organization. Geometric analysis demonstrated a similar polygon distribution in all organs with the greatest variability in the heart epithelia. Notably, the normal liver and inflated lung demonstrated the largest average cell surface area (*p* < 0.01). In lung epithelia, characteristic wavy or interdigitated cell boundaries were observed. The prevalence of interdigitations increased with lung inflation. To complement the geometric analyses, the epithelia were converted into a network of cell-to-cell contacts. Using the open-source software EpiGraph, subgraph (graphlet) frequencies were used to characterize epithelial organization and compare to mathematical (Epi-Hexagon), random (Epi-Random) and natural (Epi-Voronoi5) patterns. As expected, the patterns of the lung epithelia were independent of lung volume. In contrast, liver epithelia demonstrated a pattern distinct from lung, heart and bowel epithelia (*p* < 0.05). We conclude that geometric and network analyses can be useful tools in characterizing fundamental differences in mammalian tissue topology and epithelial organization.

## Introduction

Epithelia form sheets of cells as monolayers that line the external surface of visceral organs. Including the multilayered cells in the skin, epithelia constitute the largest and most important barrier against the external environment (Steed et al., 2010). The barrier function in visceral organs is achieved with a single layer of densely packed cells connected by a family of integral membrane proteins (claudins) that interact with scaffolding proteins and other membrane molecules (Otani and Furuse, 2020). These surface proteins and cell membranes combine to maintain an interface semipermeable to water and nutrients, but impermeable to pathogens and toxins (Tsukita et al., 2019).

Dense epithelial cell packing creates polygonal cell geometries characterized by edges that are the junctions between 2 cells and vertices that reflect the contact between three or more cells. The regulation of cell shape—as reflected by the polygonal cell geometry—appears to be critical to morphogenesis. In the embryo, constriction of the apical epithelial surface during gastrulation results in invagination (Mikawa et al., 2004; Rozbicki et al., 2015). More elaborate shape changes result in the formation of grooves and tubes and other complex structures (He et al., 2014). Importantly, the distribution pattern of cell shape is a process that drives tissue growth and repair—epithelial geometry is not simply the consequence of optimal cell packing and minimized free energy. In *drosophila*, the transition from an ordered hexagonal pattern to one that is elongated and narrow is driven, at least in part, by cell geometry (Zallen and Zallen, 2004). Cell geometry may also reflect a higher-order organization of cells (Gosak et al., 2022). Escudero et al. has applied the principles of complex network theory to identify short- and long-range patterns in epithelial organization as well as distinguish normal and abnormal epithelial tissue (Escudero et al., 2011).

An interesting feature of mammalian visceral organ epithelia is their ability to adapt to varied and dynamic environments. Examples of dynamic epithelial layers include the surface of the ventilating lung, the contracting heart and the peristaltic bowel. Previous attempts to characterize epithelial adaptations to dynamic environments have been limited by the difficulty in studying epithelium *in situ*. In contrast to the multilayered epithelia in the skin or cornea, visceral organ epithelia are a single cell layer contoured to the organ surface. The visceral epithelia—also referred to as mesothelium—are notoriously difficult to isolate intact or image *in situ* (Salvi et al., 2021). In previous work, we have adapted a method for *in situ* labeling and epithelial isolation using the bioadhesive pectin (Liu et al., 2022). A plant-derived heteropolysaccharide, pectin entangles with the surface glycocalyx to facilitate the isolation of the epithelial layer (Servais et al., 2018; Pierce et al., 2020).

In this report, epithelial cells from the lung, heart, liver and bowel were labeled *in situ* and isolated using the plant-derived bioadhesive called pectin. The isolated epithelia were imaged, segmented and digitally recombined as large epithelial montages (up to 1,200 × 1,200 µm). The epithelial images of the lung, heart, liver and bowel were analyzed for geometric and network organization.

## Methods

Animals. Male and female mice, eight- to 10-week-old wild-type C57BL/6 (Jackson Laboratory, Bar Harbor, ME), were anesthetized before euthanasia. The care and nurturing of the animals was consistent with guidelines of the American Association for Accreditation of Laboratory Animal Care (Bethesda, MD) and approved by the Brigham and Women’s Institutional Animal Care and Use Committee.

Pectin. The high methoxyl citrus pectin used in this study was obtained from a commercial source (Cargill, Minneapolis, MN, United States of America). The characterization of the high methoxyl citrus pectin has been detailed elsewhere (Zheng et al., 2021). Briefly, the pectin was cured as a translucent film with water content of 8%–12%. The pectin powder was stored in low humidity at 25°C.

Organ harvest. After general anesthesia and euthanasia by exsanguination, a midline thoracoabdominal incision exposed the visceral organs. In sequence, the left atrium, right ventricle, and inferior vena cava were incised. A 22G olive-tipped cannula was inserted through the right ventricle into the pulmonary artery, and the lungs underwent a vascular flush of 20 cc of phosphate-buffered saline. For maximally inflated lungs, a 1-ml syringe was attached to the angiocatheter and injected with 3% agarose, low gelling temperature (Sigma-Aldrich, Saint Louis, MO, United States), at 42^°^C in the volume of average TLC. The tissue was allowed to cool until solidified prior to staining and pleural epithelial isolation.

*En face* harvest. The freshly harvested organs were placed on a gel-phase high-methoxyl citrus pectin film. After 20 s of development at room temperature, the organ was peeled off at an angle of 120° with a steady rate of 2 mm per second. A thin film of PBS was maintained on the specimen to prevent dehydration (Liu et al., 2022). The pectin was then mounted on a poly-l-lysine (Sigma Aldrich, Saint Louis, MO, United States) coated glass slide. The slide was then submerged in phosphate-buffered saline for 60 min on a shaker to allow pectin to dissolve. The slides were then washed 3 times, fixed with -20°C acetone, and mounted with DAPI-containing medium (Vector Laboratories, Burlingame, CA, United States).

Silver staining. The protocol for silver staining was described elsewhere (Liu et al., 2022). Briefly, freshly harvested lung was gently rinsed with phosphate-buffered saline and incubated in 5% d-glucose (Gibco Laboratories, Grand Island, NY, United States) for 3 min. The lung surface was treated with 0.4% silver nitrate (Sigma-Aldrich) for 30 s, submerged briefly in 5% d-glucose solution prior to before exposure to 254 nm UV light (Thermoscientific, Waltham, MA, United States of America) for 60 s.

Epithelial cell image acquisition. Large epithelial montages were digitally acquired with a Nikon Plan Apochromat × 60 objective and Nikon Eclipse TE2000 inverted epi-illumination microscope. An X-Cite (EXFO, Vanier, Canada) 120-W metal halide light source and a liquid light guide were used to illuminate the *en face* samples. The 14-bit fluorescent images were digitally recorded with an electron multiplier CCD (EMCCD) camera (C9100-02, Hamamatsu, Japan). The acquisition path was facilitated a MAC5000 controller (Ludl, Hawthorne, NY) and MetaMorph software 7.10 (Molecular Devices, Downingtown, PA United States of America). A motorized stage including auto-focus were used (Ludl) for automated montage acquisition typically greater than 1,000 cells. Image stitching, after software alignment and calibration, was used to create a seamless montage based on a 10% overlap. After meticulous manual seam inspection, the high-resolution image was transferred to a separate platform for analysis by our machine learning algorithm and watershed processing. Although rare, gaps or discontinuities in the epithelium resulted in the exclusion of all connected cells. When gaps or discontinuities comprised greater than 8% of the total surface area, the images were excluded from the analysis. When multiple montages were acquired in one animal, the data were combined into one data point.

Watershed algorithm. To provide a comparison for machine learning, watershed processing was performed. The algoithm used commands from the MetaMorph 7.10 (Molecular Devices) image analysis software. The original silver stained epithelial image was processed using a series of filters that systematically flattened background regions and reduced background noise (Liu et al., 2022). Subsequent filters sequentially smoothed dark and light objects as well as connect discontinuities in the boundary regions.

Machine learning. Several machine learning algorithms were compared to our watershed method of segmentation (Liu et al., 2022). One of the neural network algorithms was widely available as a commercial site (www.biodock.ai). The silver-stained images, acquired with light microscopy, were uploaded into the neural network platforms (Liu et al., 2022). For each organ, the model was fine-tuned with an optimized subset of images relevant to creating segmented masks corresponding to cell boundaries (Sanchez-Gutierrez et al., 2016).

Centroidal Voronoi tessellations (CVT). CVT were obtained as previously described (Sanchez-Gutierrez et al., 2016). Briefly, original Voronoi diagrams were created with random seeds that were used to generate 700 iterations for each initial image. By introducing additional randomization into the seed position, a “noisy” variation of the CVT path (CVTn) was created (Vicente-Munuera et al., 2020).

Graphlet and motif selection. Images of the visceral organs were used to create a graph of cell-to-cell contacts (Escudero et al., 2011) that served as the source for the graphlet analysis (Przulj et al., 2004; Przulj, 2007). ORCA (orbit counting algorithm), a program for graphlet identification and counting (Hočevar and Demšar, 2014), was used to extract node conformation. Excluding redundant or biologically implausible graphlets, the Graphlet degree Distribution agreement Distance (GDD) of the remaining graphlets was calculated (Vicente-Munuera et al., 2020).

Statistical analysis. For each visceral organ epithelial diagram, the GDD was calculated in EpiGraph (Epi-Hexagons, Epi-Random and Epi-Voronoi5) and used to select the closest CVTn diagram. In EpiGraph, statistical significance estimation was based a comparison with randomized images as well as the impact of the original image on CVTn variance (Vicente-Munuera et al., 2020). Multivariate analysis of variance (MANOVA), useful for testing two or more populations, can be applied to two or more dependent variables. The MANOVA test was used to test the hypothesis that the samples do not arise from the same population (Izenman, 2008).

## Results

**FIGURE 1:**
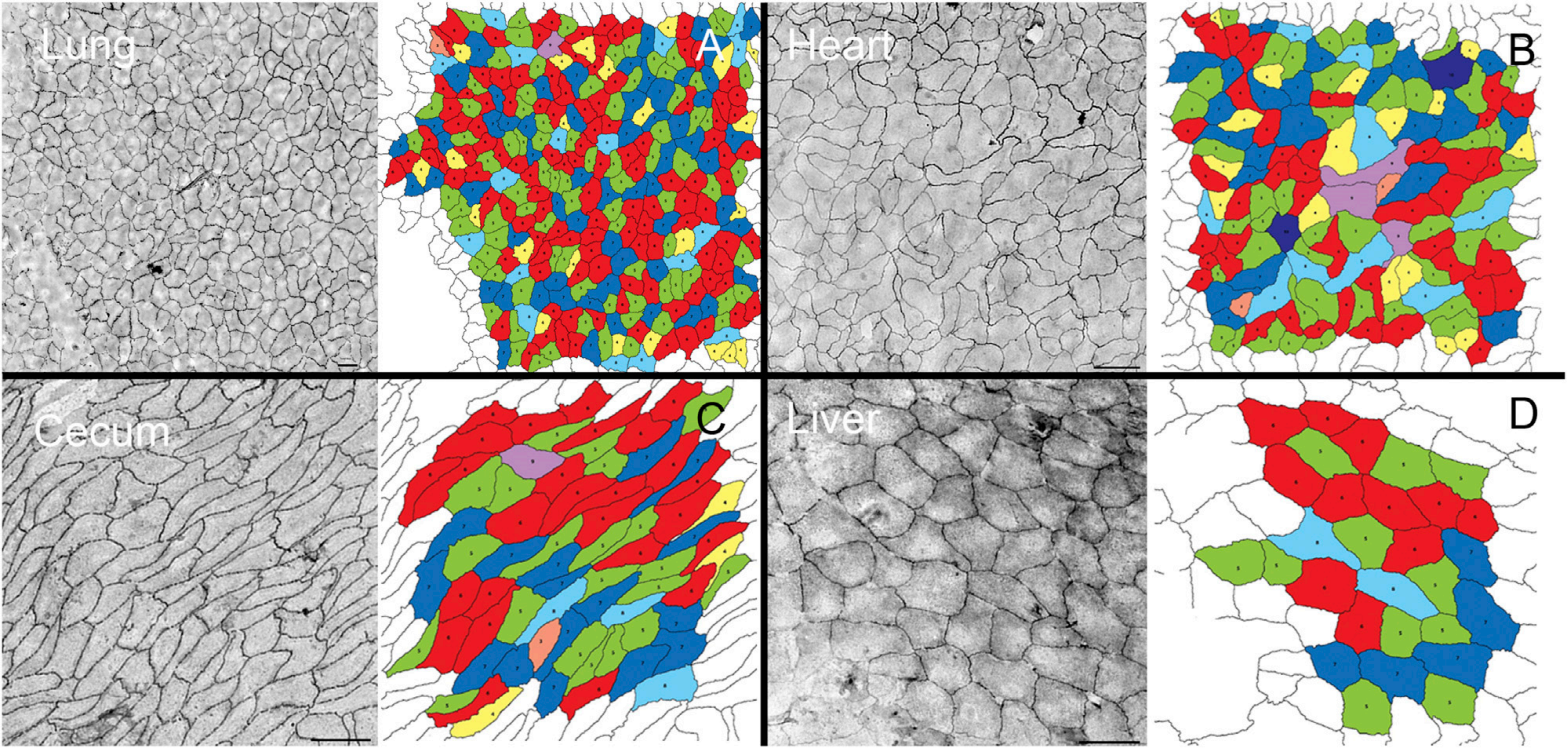
Pectin biopolymer isolation of visceral epithelium (mesothelium). A similar isolation procedure was used to harvest epithelium from freshly euthanized mice. The visceral organ epithelia including lung **(A)**, heart **(B)**, cecum **(C)**, and liver **(D)**, were isolated and cell geometry assessed by machine learning (Liu et al., 2022). In polygonal cell geometries, the edges were defined as the junctions between 2 cells, and vertices marked points of contact among three or more cells. The number of sides between vertices determine the sidedness of the polygon. The polygon distribution in the epithelial tissues was color coded: 3-sided, orange; 4-sided, yellow; 5-sided, green; 6-sided, red; 7-sided blue; 8-sided, light blue; 9-sided, purple; and 10-sided, dark purple. Bar = 200 um.

**FIGURE 2:**
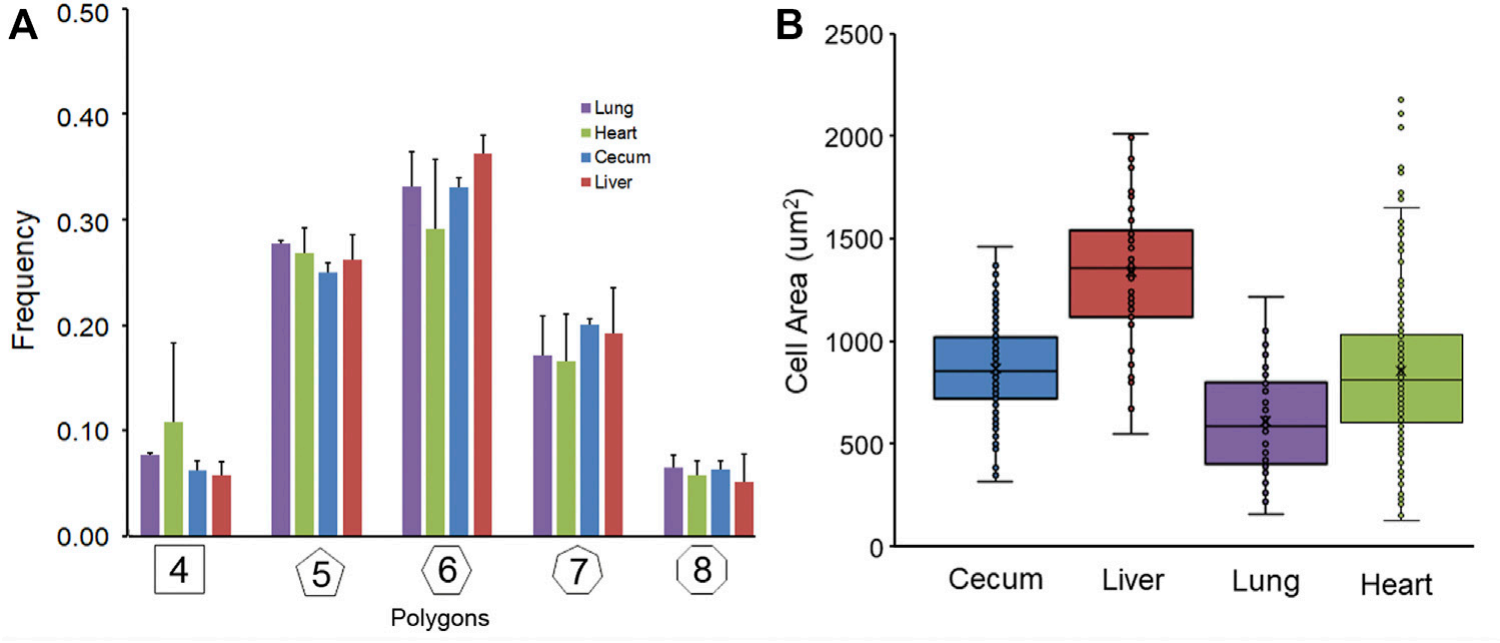
Geometric features of non-proliferating visceral organ surface epithelium. **(A)** The stained and harvested epithelia were analyzed by a machine learning algorithm previously described (Liu et al., 2022). The polygon frequency distribution was compared in images obtained from lung, heart, liver and cecum samples. Similar polygon frequencies were observed with the heart demonstrating the greatest variability. **(B)** Morphometric analysis facilitated the calculation of cell surface area. The liver cell surface area was significantly greater than the pleural epithelial cells surface analyzed at residual volume (*p* < 0 .05). Note, the lung was uninflated during epithelial harvest. The box spans the interquartile range of the replicate samples with the median marked with an X and the whiskers defining the statistical data range.

Geometric organization. After *in situ* silver staining of the organ surface, pectin was used to harvest the visceral organ epithelium. The isolated epithelium was imaged and analyzed for geometric features. Silver stained samples of lung, heart, cecum and liver demonstrated detectable cell boundaries (Figure 1). The polygon distribution of the epithelia was notable for the greatest variability in the lung and heart (Figure 2A). Notably, the expected equivalence of the numer of n-gons (*n* < 6 = *n* > 6) (Roshal et al., 2022) was not observed; the variance ranged from 5% in the cecum to 12% in the heart. The liver demonstrated the largest average cell surface area and the uninflated pleural epithelium demonstrated the smallest surface area (Figure 2B) (*p* <0 .05).

**FIGURE 3:**
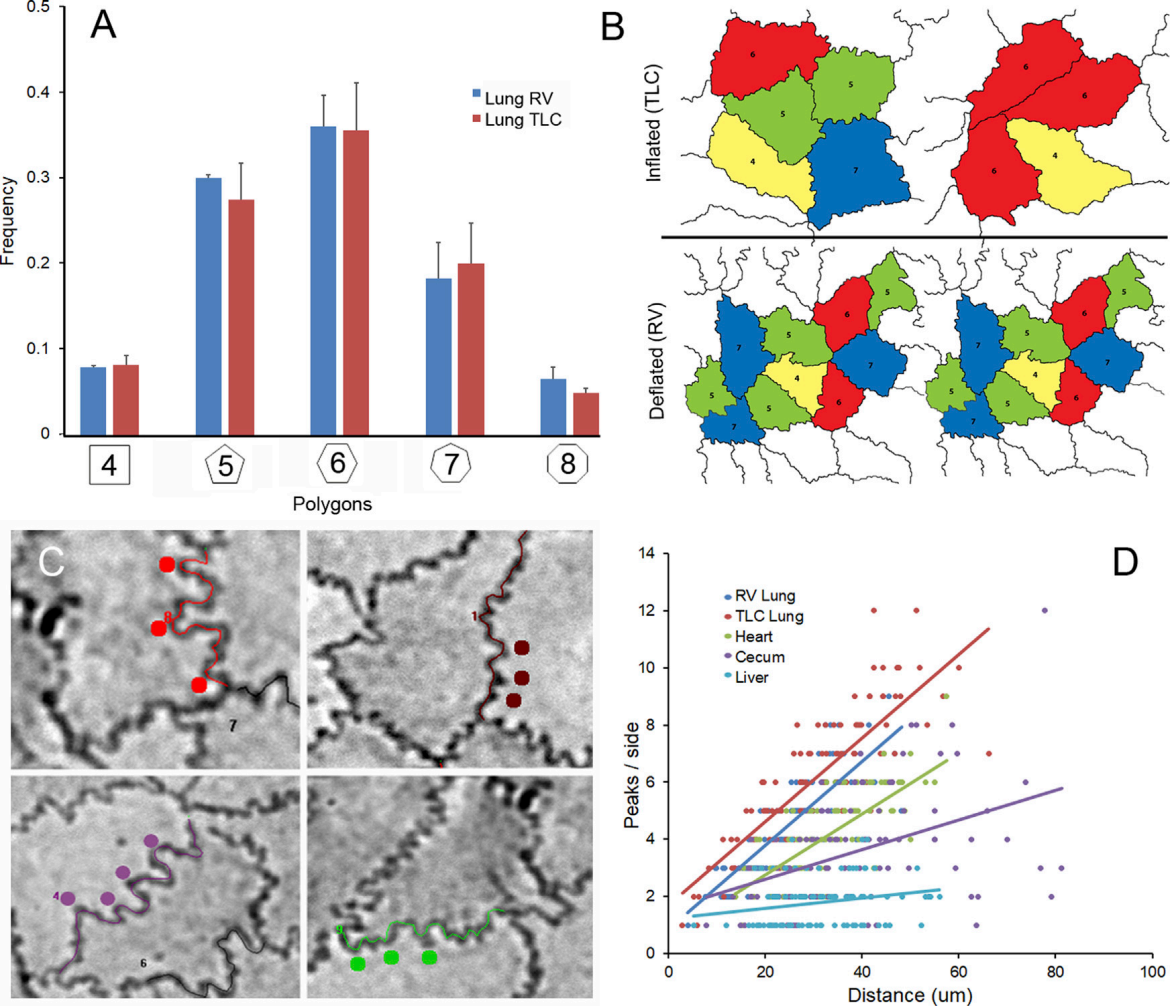
Effect of lung expansion with inhalation. **(A)** The polygon frequency distribution was compared in images obtained from lung at RV and TLC. The polygon frequency distribution was not significantly different (*p* >0 .05) between inflated (TLC) and deflated (RV)lungs. **(B)** Inflation was associated with a significant increase in cell area (*p* <0 .05). The polygon distribution in the epithelial tissues was color coded: 4-sided, yellow; 5-sided, green; 6-sided, red; 7-sided blue; 8-sided, light blue. **(C)** Both inflated and deflated lungs were associated with wavy or interdigitated cell borders (colored dots). **(D)** The number of peaks (interdigitations) were more prevalent in the lung samples and increased linearly with increased side length: lung (TLC) *R* ^2^ = 0.687, lung (RV) *R* ^2^ = 0.671, heart *R* ^2^ = 0.542, cecum *R* ^2^ = 0.185 and liver *R* ^2^ = 0.052.

**FIGURE 4:**
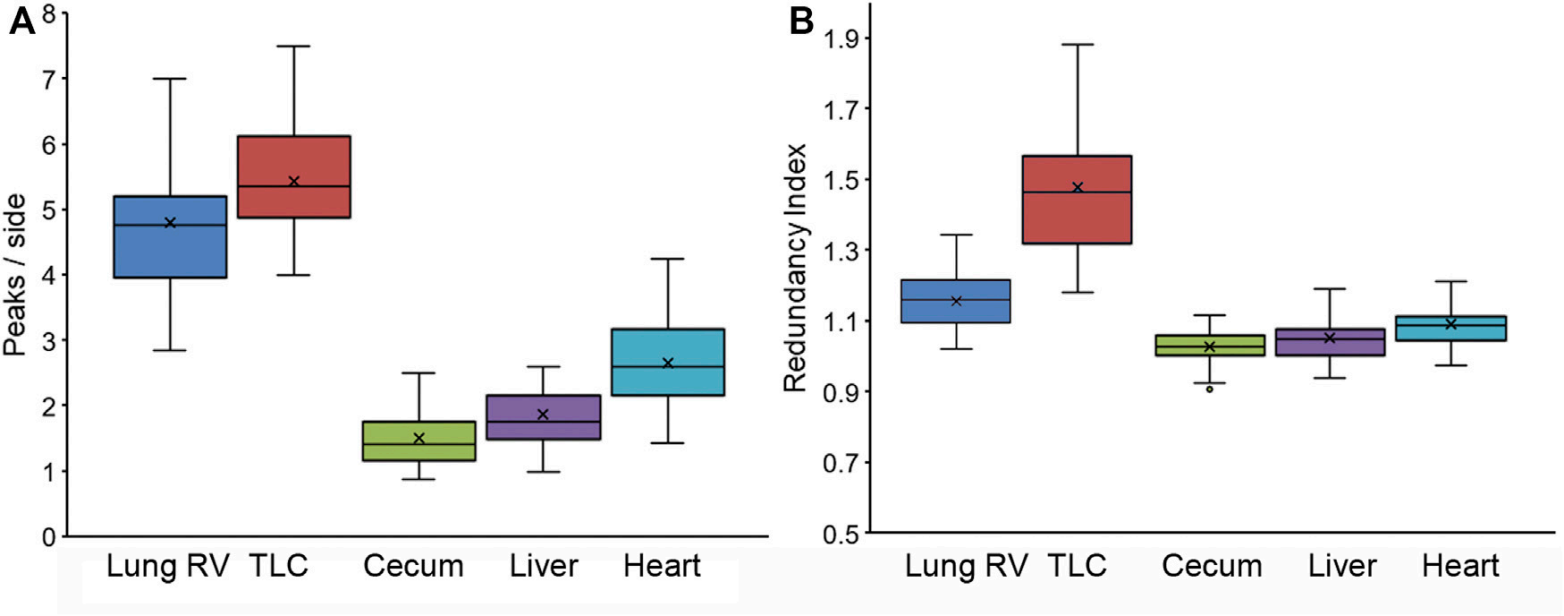
Characterization of cell boundary interdigitations using two methods. **(A)** The mean number of interdigitations per side was independently quantified by a minimum of two observers. **(B)** The redundancy index was defined as the length of the traced interdigitated boundary compared to the theoretical minimum distance between vertices. The redundancy index value was expressed as a ratio with 1.0 reflecting a straight border without interdigitation.In both graphs, the box spans the interquartile range with the median marked with an X in the whiskers defining the data range.

Pleural epithelial organization. The pleural epithelial surface area has been shown to increase more than 50% with inflation from residual volume (RV) to total lung capacity (TLC) (Liu et al., 2022). To characterize pleural epithelial geometry, the epithelial cells were harvested from the two epithelial cell extremes: RV and TLC. As expected, the polygon distribution was similar at RV and TLC (Figure 3A). Cell size, however, was significantly increased with inflation (Figure 3B). The mean cell size of the inflated lung and liver epithelial cells was significantly greater than heart or spleen epithelial cells (*p* < 0 .01). Also noted were wavy or interdigitated cell boundaries (Figure 3C). The number of interdigitations increased commensurate with border length (Figure 3D). To quantify the interdigitations, we used two methods: counting the interdigitations (Figure 4D) and measuring the border redundancy (Figure 4D), Both methods demonstrated a striking prevalence in the lung compared to other visceral organs. Notably, the number of interdigitations increased with lung expansion (Figure 4D).

**FIGURE 5:**
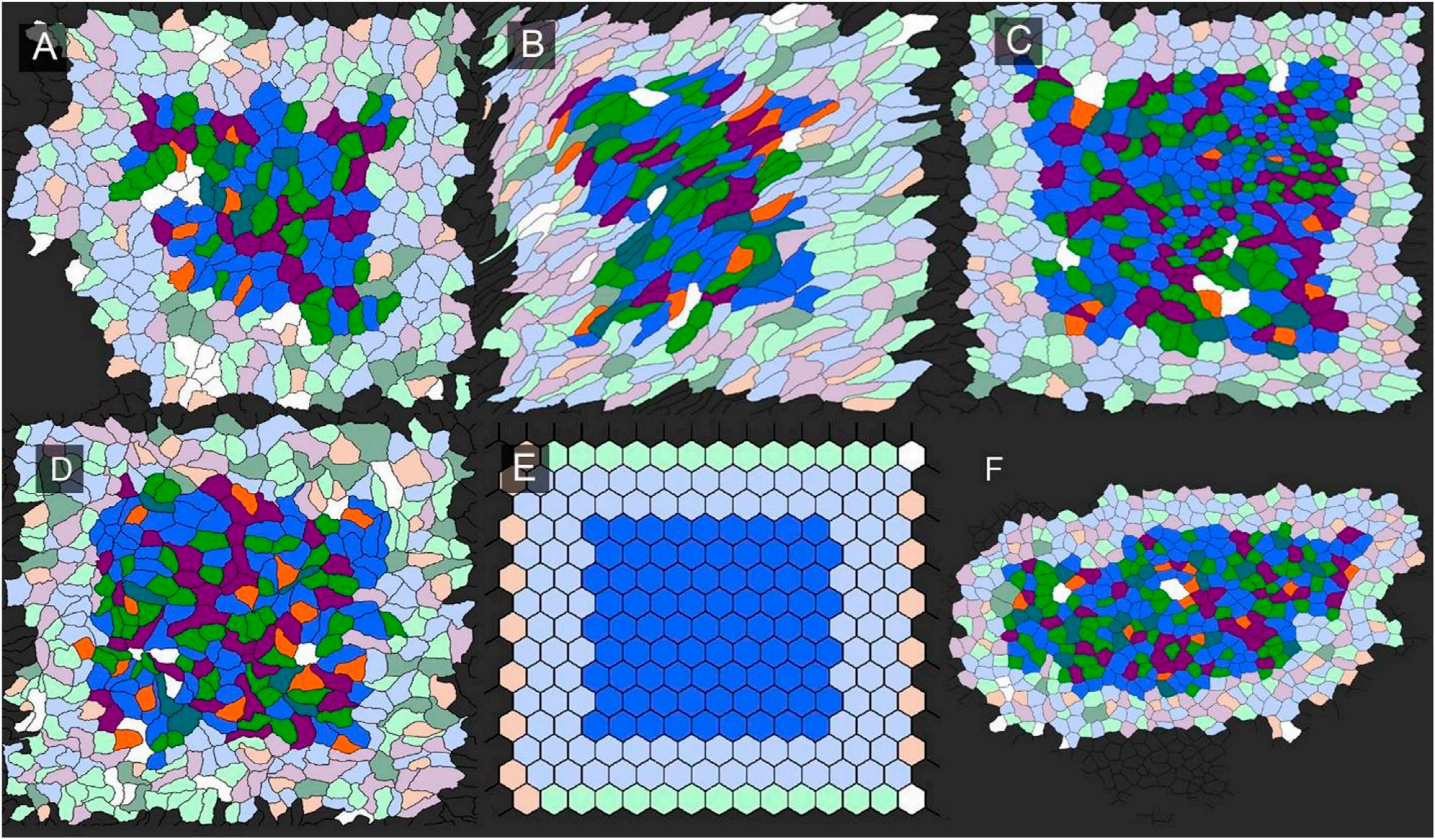
Characterization of network graphlets in epithelial tissues. An illustration of the analysis is shown for lung, cecum, heart, liver and liver. A mathematical tessellation in the form of hexagons was included for comparison. Borders around each region of interest reflects the requirement for a distance of four edges to quantify graphlet frequency. The visceral organs included **(A)** heart, **(B)** cecum, **(C)** lung, **(D)** heart, **(E)** hexagon, and **(F)** liver.

**FIGURE 6:**
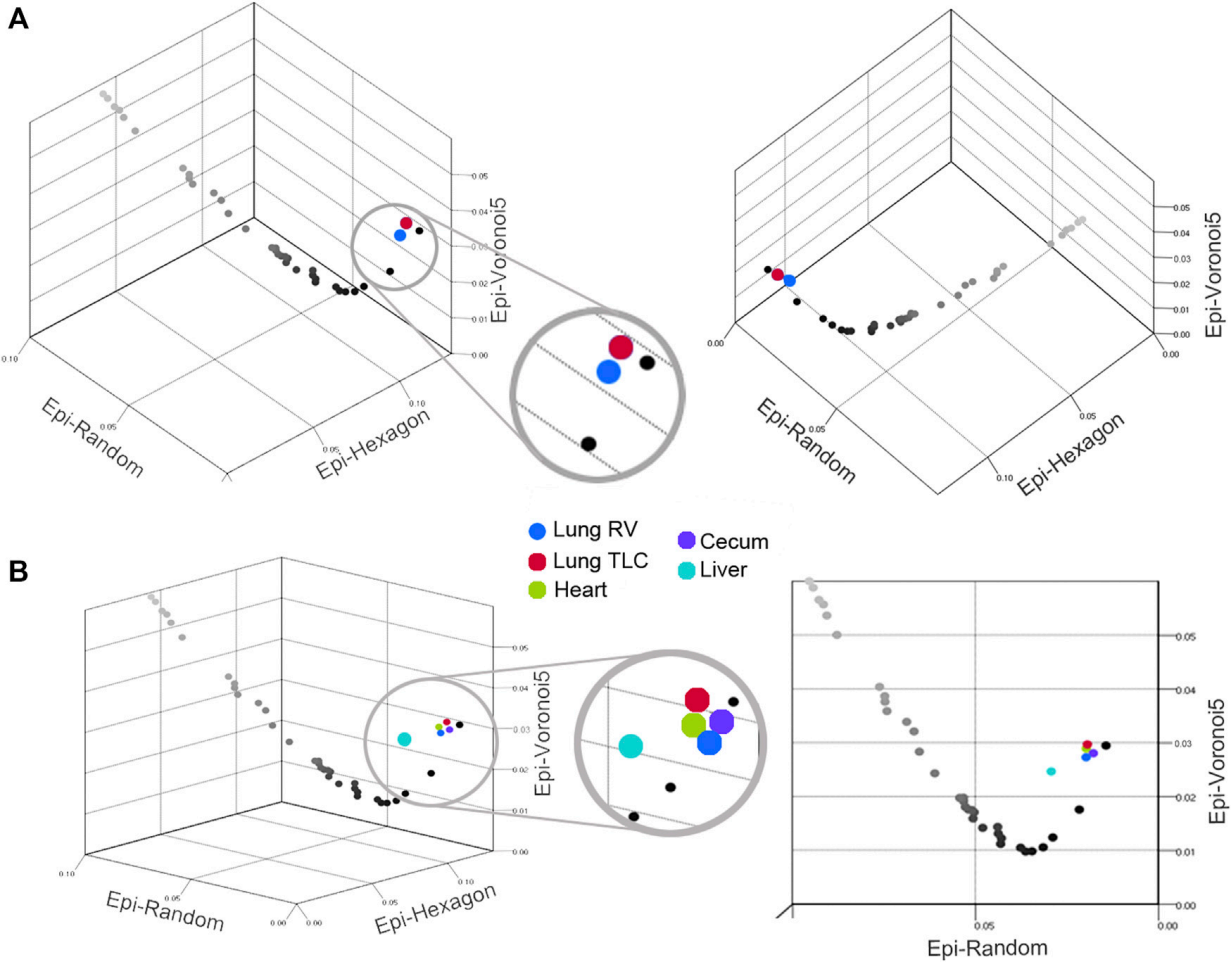
Scatter plot of the Graphlet degree Distribution agreement Distance (GDD) values for epithelia from the lung **(A)** and all four visceral organs **(B)**. The plots demonstrate the GDD values (Epi-Hexagons, Epi-Random and Epi-Voronoi5) of individual biological tessellations: blue, lung RV; red, lung TLC; green, heart; purple, cecum and teal, liver. The plots displayed the CVTn scale from iteration 1 (black) to iteration 700 (light gray). The liver was the only statistically dissimilar epithelium (teal, *p* < 0 .05).

Network organization. To examine the relevance of Graph Theory as a tool for characterizing epithelial organization, segmented images from the visceral organs were analyzed by the open-source software EpiGraph (Vicente-Munuera et al., 2020). EpiGraph analyzed segmented images for graphlet frequency (Figure 5). The visceral organs were compared to mathematical (Epi-hexagon), random (Epi-random) and naturally-occurring (Epi-Voronoi5) patterns (Vicente-Munuera et al., 2020). Using the EpiGraph software, the lung epithelial organization at RV and TLC were similar (Figure 6). Both were plotted on the CVTn path indicating organizational similarity (Figure 6A). In contrast, the liver epithelium was plotted outside of the CVTn path suggesting a potentially distinctive organizational structure. Although the liver analysis trended toward significant differences from the lung, heart and lung epithelial organization, MANOVA was marginally significant (*p* < 0 .05).

## Discussion

In this report, we used *in situ* labeling and *en face* harvesting to examine the geometric and network organization of non-proliferating visceral organ epithelia. Our analysis led to several empirical observations. First, visceral organ epithelia demonstrated a characteristic cell geometry distribution independent of cell size. Second, pleural epithelia demonstrated a 50% increase in average cell surface area with lung inflation. Third, the pleural epithelial cells demonstrated a wavy or interdigitated border that was enhanced with lung expansion. Fourth, the network organization of the liver epithelia was distinct from the organization of the lung, heart and bowel. We conclude that geometric and network analyses can be useful tools in characterizing fundamental differences in mammalian tissue topology and epithelial organization.

The essential features of epithelial construction are remarkably preserved among metazoa. Prior studies of such diverse epithelia as *Drosophila*, *Hydra*, *Xenopus* and *Dryopteris* have demonstrated remarkable consistency (Gibson and Gibson, 2009) as well as some notable exceptions (Knust and Bossinger, 2002). Invariably, hexagons are the modal polygon class (∼40%) followed by pentagons (∼25%) and heptagons (∼20%) (Zallen and Zallen, 2004) (Korn and Spalding, 1973). This relative frequency distribution was reflected in our visceral organ epithelia analysis. The difference in our data was at the tails of the distribution. In most epithelial distributions, the reported number of 4-sided and 8-sided cells has been 1%–5%; in our data, the number was consistently 5%–8%.

First studied in the 1920 s (Lewis, 1926; 1928), the topology of the cell borders provides potential insights into epithelial organization. Assuming some basic features of epithelial cell division, recent theories have proposed the hypothesis of topological invariance (Gibson et al., 2006). This hypothesis predicts that the distribution of polygon types must be balanced; that is, the number of *n*-gons *n* < 6 must equal the number of *n* > 6. Roshal and others have proposed that this relationship provides an estimate of the error in an experimental system (Roshal et al., 2022). An interesting finding in our study was the persistent magnitude of the variance at approximately 10%--a magnitude substantially larger than variance observed *in vitro* (Roshal et al., 2022). Of course, this variance may reflect the methodologic challenges of studying mammalian tissues (Liu et al., 2022). Alternatively, the variance may reflect junctional rearrangements caused by unique features of mammalian epithelia such as cell motility, epithelial-mesenchymal transitions, cell jamming and cell death. In either case, the distribution of polygon types is an important consideration in future work.

We analyzed the tissue organization of packed visceral organ epithelia using the open-source EpiGraph software (Vicente-Munuera et al., 2020). EpiGraph evaluated the organizational similarities of epithelial tissues using common properties explored with Voronoi tessellations and Graph Theory. In mathematical Voronoi tessellations, the data points are partitioned into Voronoi cells in planar space. The iterative optimization of the Voronoi partitions toward a theoretical Centroid of Voronoi Tessellation (CVT), such as the Lloyd algorithm (Lloyd, 1957), creates a path that can be used as a relative scale to compare packed epithelial tissues as well as an absolute scale to compare natural and mathematical tessellations. Applied to the visceral organ epithelia in our work, the CVT path was a useful measure of tissue organization.

An EpiGraph’s Graph Theory application, the packed epithelial cells were converted into a network of cell-to-cell contacts. The features and patterns of the graph were subdivided into subgraphs (graphlets). By analyzing subgraph frequency, EpiGraph software has been used to quantify differences between complex systems (Escudero et al., 2007; Escudero et al., 2011; Sanchez-Gutierrez et al., 2013; Sanchez-Gutierrez et al., 2016). Although a comparison of two large networks is computationally infeasible (Przulj, 2007), the relatively small, segmented images from real epithelial layers were readily analyzed and compared. The graphlet approach to capturing the topology of epithelial tissue provided not only a quantitative measure of the differences between epithelial networks, but also the potential for insights into mechanisms of network growth and repair.

An intriguing observation was the silver stain demonstrating wavy and interdigitated cell borders. Anatomic features responsible for the interdigitated lines are unclear; however, the wavy lines were prominently linked to organ expansion. The relatively static liver epithelium demonstrated few interdigitations, whereas the ventilating lung epithelium demonstrated a significant number of interdigitations. Furthermore, lung inflation from RV to TLC was associated with an increased number of interdigitations. A potential explanation is that the change in organ volume—whether it reflects lung inflation, cardiac contraction or bowel peristalsis—involves cell surface area in the apical Z-dimension that is contributed to the X-Y dimension during organ expansion. The additional cell surface area, constrained by tight junctions, leads to interdigitated cell borders. Another possibility is that the border variations between cells reflects cytoskeletal dynamics between neighboring cells. Reminiscent of the jigsaw puzzle appearance of pavement cells in plants (von Wangenheim et al., 2017), the convex and concave borders in the leaf *epidermis* have been associated with compositional heterogeneity at the cell-cell interface (Majda et al., 2017).

A common understanding of cellular physiology is that the cell size is not fixed but responds to external factors (Miettinen et al., 2017). Structural and metabolic plasticity is presumably important for adaptation to changing environments. Here, we found that cell surface area in the planar dimension appeared to reflect environmental changes at the organ surface layer. The liver, an organ associated with relatively little change at the organ surface, demonstrated a large mean cell area and the least variability in cell surface area. In contrast, the lung, an organ associated with significant surface changes during ventilation, demonstrated both the smallest (RV) and the largest (TLC) cell surface area. The compressed time scale of normal ventilation (many breaths/minute) suggests that the observed changes in pleural cell surface area do not reflect changes in cell volume, but rather dynamic changes in cell shape. In our future work, we will explore the cytoskeletal and membrane dynamics during organ expansion.

## Data Availability

The raw data supporting the conclusion of this article will be made available by the authors, in accordance with standard academic practice.

## Abbreviations

CVT, Centroidal Voronoi Tesselation; CVTn, noisy Centroidal Voronoi Tesselation; GDD, Graphlet degree Distribution agreement Distance; RV, residual volume; TLC, total lung capacity.
